# Integrated metabolomics and transcriptomics insights on flavonoid biosynthesis of a medicinal functional forage, *Agriophyllum squarrosum* (L.), based on a common garden trial covering six ecotypes

**DOI:** 10.3389/fpls.2022.985572

**Published:** 2022-09-20

**Authors:** Tingzhou Fang, Shanshan Zhou, Chaoju Qian, Xia Yan, Xiaoyue Yin, Xingke Fan, Pengshu Zhao, Yuqiu Liao, Liang Shi, Yuxiao Chang, Xiao-Fei Ma

**Affiliations:** ^1^Key Laboratory of Stress Physiology and Ecology in Cold and Arid Regions, Department of Ecology and Agriculture Research, Northwest Institute of Eco-Environment and Resources, Chinese Academy of Sciences, Lanzhou, China; ^2^College of Resources and Environment, University of Chinese Academy of Sciences, Beijing, China; ^3^Faculty of Environmental Science and Engineering, Shanxi Institute of Science and Technology, Jincheng, China; ^4^Key Laboratory of Eco-Hydrology of Inland River Basin, Northwest Institute of Eco-Environment and Resources, Chinese Academy of Sciences, Lanzhou, China; ^5^Marsgreen Biotech Jiangsu Co., Ltd., Haian, China; ^6^Agricultural Genomics Institute at Shenzhen, Chinese Academy of Agricultural Sciences, Shenzhen, China

**Keywords:** *Agriophyllum squarrosum*, sandrice, flavonoids, transcriptome, metabolome, common garden, isorhamnetin-3-glycoside, isorhamnetin

## Abstract

*Agriophyllum squarrosum* (L.) Moq., well known as sandrice, is an important wild forage in sandy areas and a promising edible and medicinal resource plant with great domestication potential. Previous studies showed flavonoids are one of the most abundant medicinal ingredients in sandrice, whereby isorhamnetin and isorhamnetin-3-glycoside were the top two flavonols with multiple health benefits. However, the molecular regulatory mechanisms of flavonoids in sandrice remain largely unclear. Based on a common garden trial, in this study, an integrated transcriptomic and flavonoids-targeted metabolomic analysis was performed on the vegetative and reproductive periods of six sandrice ecotypes, whose original habitats covered a variety of environmental factor gradients. Multiple linear stepwise regression analysis unveiled that flavonoid accumulation in sandrice was positively correlated with temperature and UVB and negatively affected by precipitation and sunshine duration, respectively. Weighted co-expression network analysis (WGCNA) indicated the *bHLH* and *MYB* transcription factor (TF) families might play key roles in sandrice flavonoid biosynthesis regulation. A total of 22,778 differentially expressed genes (DEGs) were identified between ecotype DL and ecotype AEX, the two extremes in most environmental factors, whereby 85 DEGs could be related to known flavonoid biosynthesis pathway. A sandrice flavonoid biosynthesis network embracing the detected 23 flavonoids in this research was constructed. Gene families *Plant flavonoid O-methyltransferase* (*AsPFOMT*) and *UDP-glucuronosyltransferase* (*AsUGT78D2*) were identified and characterized on the transcriptional level and believed to be synthases of isorhamnetin and isorhamnetin-3-glycoside in sandrice, respectively. A trade-off between biosynthesis of rutin and isorhamnetin was found in the DL ecotype, which might be due to the metabolic flux redirection when facing environmental changes. This research provides valuable information for understanding flavonoid biosynthesis in sandrice at the molecular level and laid the foundation for precise development and utilization of this functional resource forage.

## Introduction

Plant secondary metabolites play important roles in mediating plant responses to environmental factors (Yang et al., 2012). As the largest group of secondary metabolites in plants, flavonoids are regarded as one of the key adaptations to additional abiotic stresses of early land plants during the transition toward a non-aquatic lifestyle in land colonization (Albert et al., 2018; Wen et al., 2020). Though its mechanism of anti-oxidative activity remains unclear to some extent, flavonoids were reported as defense compounds in plants (Hernández et al., 2009). Excessive accumulation of flavonoids provides extensive stress tolerance *via* eliminating reactive oxygen species (ROS), and responses to biotic stresses (like pathogen infection and insect predation) as well as nearly all kinds of abiotic stresses, including saline-alkali stress, dehydration, heat, cold, high light intensity, UV radiation, etc. (Nakabayashi et al., 2014; Nakabayashi and Saito, 2015; Ma et al., 2020; Böttner et al., 2021; Dong and Lin, 2021; Xu et al., 2022). On the other hand, the past few decades witnessed growing evidence for the multiple benefits of flavonoids on human wellbeing as plant-derived nutraceuticals. Flavonoids were proved to be anti-inflammatory agents and anti-oxidants, and therefore a therapeutic intervention for a variety of chronic diseases and metabolic syndromes (Galleano et al., 2012; Yi, 2018), including cancer (Le Marchand, 2002; Burkard et al., 2017), cardiovascular diseases (Hodgson and Croft, 2010; Fusi et al., 2020), diabetes (Hussain et al., 2020; Proença et al., 2022), obesity (Rufino et al., 2021), respiratory system diseases (Somerville et al., 2016), and neurodegenerative diseases, e.g., Alzheimer’s and Parkinson’s diseases (Solanki et al., 2015; Spagnuolo et al., 2018; Tahir et al., 2021).

Flavonoids have been conservatively estimated to comprise over 8,000 metabolites and could be categorized into six subclasses according to different substituting positions on the common diphenylpropane (C6-C3-C6) backbone, e.g., flavones, flavonols, flavanones, flavanols, anthocyanidins, and isoflavones (Dong and Lin, 2021). Flavonoid biosynthesis in plants begins with the general phenylpropanoid pathway (GPP), a three-step enzymatic reaction catalyzed by phenylalanine ammonia lyase (PAL), cinnamate 4-hydroxylase (C4H), and p-coumaroyl coenzyme A ligase (4CL), generating p-coumaroyl-CoA (Vogt, 2010). The two main outlets of the phenylpropanoid pathway, lignin, and flavonoid, branch off from here and are catalyzed by Hydroxycinnamoyl-CoA shikimate/quinate hydroxycinnamoyl transferase (HCT) and chalcone synthase (CHS), respectively. CHS catalyzes the first step in sandrice flavonoid biosynthesis to synthesize chalcone and directs the metabolic flux to flavonoid metabolism. Next, chalcone isomerase (CHI) acts on chalcones and generates flavanones, for example, naringenin. Then, a series of successive enzymatic reactions synthesize different classes of flavonoid compounds, whereby methylation and glycosylation are two prevalent processes (Shelton et al., 2012; Wang S. et al., 2019).

Plants are the only natural resource of flavonoids (Hernández et al., 2009). Due to their resilience to local marginal environments, remarkable biodiversity, and multiple dietary benefits, pseudocereals have greater potential as sources of natural flavonoid exploitation and utilization. Sandrice [*Agriophyllum squarrosum* (L.)] is an edible and medicinal resource psammophyte of Chenopodiaceae, which is widely distributed in the vast arid and semi-arid sandy lands in Central Asia, the Caucasus, Mongolia, Siberia, and Northern China (Qian et al., 2016). Sandrice is a pioneer species on mobile sands, being able to adapt to the cruel environment in deserts like extreme temperature, dehydration, and sand burial, and was proved to have a wide range of genetic and phenotypic variation among different populations, which was derived from local adaptation to their original marginal habitats (Yin et al., 2016; Zhao et al., 2017, 2022; Qian et al., 2021). Although this underutilized feed crop has not been domesticated, the seeds of sandrice have a long consumption history and are rich in essential amino acids, crude fiber, polyunsaturated fatty acids, etc., and tender stems and leaves of sandrice are wild feedings for livestock in the sandy areas, making it an ideal functional food and natural feed crop (Li et al., 1992; Chen et al., 2014; Wang Q. et al., 2019; Han et al., 2021; Zhao et al., 2021). Additionally, the aerial part of sandrice was documented as a medicinal plant for kidney inflammation, dyspepsia, antipyretic, and analgesic treatment in Traditional Chinese Medicine (TCM) as well as in Mongolian medicine. Recent research on sandrice not only elucidated its potential as a feed additive for improving the growth performance of livestock (Liang et al., 2021), but also clarified the efficacy of human diabetes treatment and liver protection (Saqier et al., 2019; Bao et al., 2020). Moreover, our previous studies provided evidence of the abundance and diversity of multiple active pharmaceutical ingredients in sandrice, such as terpenoids, phenolic acids, alkaloids, and especially flavonoids (Yin et al., 2018; Zhou et al., 2021a,b). Thus, the plant is of great domestication potential and capacity of offering dietary and medical benefits to human health, in the context of global climate change and continuous consumption expansion.

Our latest research showed that isorhamnetin is the most abundant flavonoid among a variety of ecotypes of sandrice (Zhou et al., 2021a). Isorhamnetin, and its derivate, isorhamnetin-3-glycoside, were proved to have multiple health benefits, including anti-virus (Urzainqui and Carrasco, 1989), cardiovascular and cerebrovascular protection (Gong et al., 2020), anticancer (Saud et al., 2013; Hu et al., 2019; Park et al., 2019), anti-inflammation (Yang et al., 2013; Xu et al., 2022), detoxification (Kim et al., 2021), and could be a prescription of diabetes (Matboli et al., 2021; Kalai et al., 2022). Together with a few other flavonoids, isorhamnetin was recommended as a dietary supplement to enhance immunity and relieve inflammation of the respiratory system, in the context of the COVID-19 global pandemic (Li et al., 2021). Isorhamnetin, or 3-methylquercetin, is formed *via* the methylation of quercitrin at the 3′-OH and catalyzed by S-adenosyl-L-methionine (SAM) dependent O-methyltransferase family (OMTs, Xie et al., 2022). Plant OMTs can be divided into families PlOMTI (caffeoyl-CoA O-methyltransferase, CCoAOMT) and PlOMTII (caffeic acid 3-O-methyltransferase, COMT) according to their molecular weights and Mg^2+^-dependency, or categorized into five subfamilies based on substrate preference (Liu et al., 2019). Moreover, three novel OMTs that had high similarity with CCoAOMT, while presented substrate preference in flavonoids was identified in *Mesembryanthemum crystallinum*, and formed a subclade in the CCoAOMT family and designated as PFOMT or CCoAOMT-like (Ibdah et al., 2003). A few studies provided experimental evidence of members in the PFOMT subclade being capable of specifically catalyzing the methylation of quercetin to form isorhamnetin in *Mesembryanthemum crystallinum* (Ibdah et al., 2003), *Serratula tinctoria* (Huang et al., 2004), *Glycine max* (Kim et al., 2006), *Citrus reticulata* (Liu et al., 2020), *Prunus persica* (Xie et al., 2022), etc. However, there is currently no report available on systematic analyses of PFOMT in sandrice or its closely related species, which hinders the precise development of this valuable resource plant.

In our previous studies, differentially accumulation of multiple flavonoids was detected between high- and middle-altitudinal sandrice ecotypes *in situ* (data unpublished). After transplanting to a common garden trial in a middle-altitudinal location, flavonoids were diversified and enriched among 14 ecotypes of sandrice along altitudinal gradients, leading us to the deduction that the enrichment of flavonoids in the high-altitude populations of sandrice was a consequence of physiological response to environmental stresses other than of genetic differentiation involved in local adaptation to the high altitude (Zhou et al., 2021a). To provide further evidence to this hypothesis on the transcriptional level, and to elucidate the underlying molecular basis and regulatory modules of characteristic flavonoid biosynthesis, especially that of isorhamnetin and isorhamnetin-3-glycoside in sandrice, here, based on a common garden trial, we expanded our previous research by integrated analysis combined metabolomics and transcriptomics of six sandrice ecotypes, whose original habitats covering multiple environmental factors gradients, across two developmental stages. Multivariance regression analysis provides solid evidence on the question of whether and how sandrice flavonoid accumulation involves in local adaptation to their original habitats, as a complement and revise to univariate linear regression. Weighted co-expression network analysis (WGCNA) and subsequent hub gene digging were conducted to discover possible regulatory molecules, e.g., transcription factors (TF), regarding flavonoid biosynthesis. Besides, one of our aims was to identify synthases of isorhamnetin and isorhamnetin-3-glycoside in sandrice, for their abundance and potential health benefits. In brief, this research provided preliminary but valuable support for flavonoid biosynthesis in sandrice, and pave the way for the precise development of this resource plant in the health industry.

## Materials and methods

### Plant growth, common garden trial, and sample collection

**FIGURE 1:**
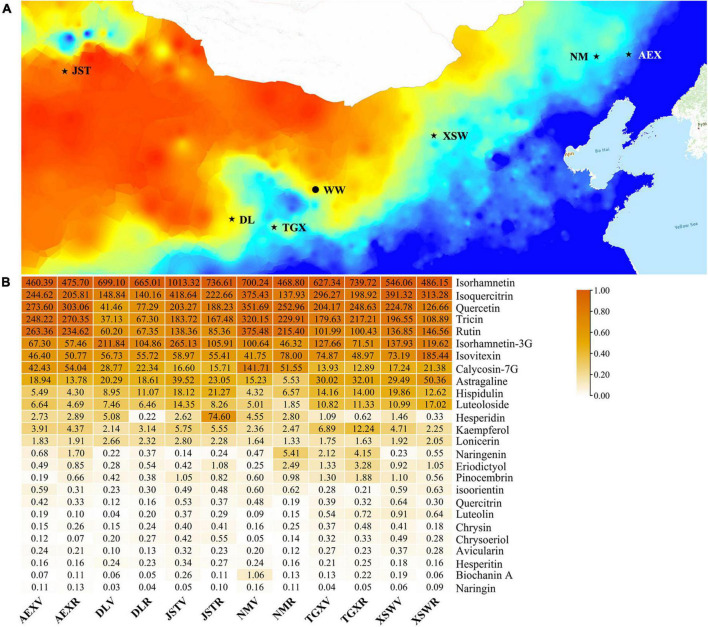
Map of samples collected and the contents of flavonoids in the 12 samples. **(A)** Original habitats of the six ecotypes and the location of the common garden trials are marked as stars and dots, respectively. The base map showed the gradient of annual precipitation, from high (blue) to low (red). **(B)** The number in each cell is the average content in μg/g. Modified from Zhou et al. (2021a). Stars are locations of original habitats of each sample.

Seeds of six sandrice ecotypes, AEX, DL, JST, NM, TGX, and XSW, were collected originally from their habitats, which cover multiple environmental factors gradients in Northern China deserts and sandy lands (Figure 1A and Supplementary Table 1). Seeds were planted in a common garden trial conducted in natural environmental conditions nearby the City of Wuwei, Gansu Province (Northwest China, Tengger Desert, 37°54′10.98”N, 102°54′4.2”E, 1,530 m) in April 2019. Mature leaf samples from all the six ecotypes were collected in June (vegetative growth stage of sandrice) and August (reproductive growth stage of sandrice), and were assigned as V and R, respectively. Pooled leaves from the same developmental stage of at least two or more individuals of the six ecotypes were immediately frozen in liquid nitrogen, preparing for ribonucleic acid (RNA) extraction.

### Ribonucleic acid extraction and RNA-seq library construction

RNA sequencing was performed to understand the molecular mechanism of flavonoid biosynthesis in sandrice. Total RNA was extracted with the RNAprep Pure Plant Plus kit (TIANGEN, Beijing, China) and treated with RNase-free DNase I (TIANGEN, Beijing, China), following the manufacturer’s guidebooks. RNA concentration and purity were measured using NanoDrop 2000 (Thermo Fisher Scientific, Wilmington, DE). RNA integrity was assessed using the RNA Nano 6000 Assay kit of the Agilent Bioanalyzer 2100 system (Agilent Technologies, CA, United States). Twelve RNA sequencing samples were generated using NEBNext^®^ Ultra™ Directional RNA Library Prep Kit for Illumina^®^ (NEB, Ipswich, MA, United States) following the manufacturer’s recommendations. Library quality was assessed by the Agilent Bioanalyzer 2100 system. After cluster generation, the library preparations were sequenced on an Illumina Hiseq Xten platform (Illumina Inc., San Diego, CA, United States) and paired-end reads were generated. The raw sequencing files of transcriptomic data will be available in the NCBI SRA database with accession number PRJNA853545.

### Quality control, assembly, and gene functional annotation

Clean data were obtained by removing reads containing adapter, poly-N, and low-quality paired-end raw reads. All clean data with high quality were subsequently mapped to sandrice reference genome (unpublished data) and formed a joint transcript using HISAT2-Stringtie pipeline with default parameter. To provide insight into the functions of newly identified sandrice transcripts, the annotation for all unigenes was performed by running BLAST against the following databases: Nt (NCBI non-redundant nucleotide sequences); Swiss-Prot (A manually annotated non-redundant protein sequence database); InterProt (The InterPro protein families and domains database); TrEMBL (Atlas of Protein Sequence and Structure); GO (Gene Ontology database); and TAIR (A resource for integrated Arabidopsis data) with an *E*-value threshold of 1e-5 as significant hits.

### Multivariance stepwise regression analysis

Multivariance stepwise regression analysis was conducted by IBM SPSS Statistic 25 (SPSS Inc., Armonk, NY, United States) to determine the main environmental factors affecting flavonoid accumulation in sandrice. The 22 environmental climatic factors from the original habitats of the six ecotypes (Supplementary Tables 1, 2) were extracted by ArcGIS 10.2 (ESRI Inc., California, United States) and DIVA-GIS 7.5.0.

### Metabolic profiling and differentially accumulated flavonoids analysis

Details of flavonoid-targeted metabolic profiling were listed in our previous research (Zhou et al., 2021a). OPLS-DA analyses for differentially accumulated flavonoids (DAF) identification between different developmental stages within an ecotype and across ecotypes were performed using SIMCA 13.0 (Umetrics, Umeå, Sweden) and OmicShare tools.^1^

### Identification and biological function analysis of differentially expressed genes

Differential expression analysis between developmental stages and among ecotypes was performed using the EdgeR package (3.32.1). Genes met an adjusted *P*-value < 0.05 and the absolute value of log_2_(fold change) ≥ 1 were assigned as DEGs. Gene Ontology (GO) and Kyoto Encyclopedia of Genes and Genomes (KEGG) enrichment analysis of DEGs were implemented by the clusterProfiler R package and OmicShare tools, using hypergeometric testing to find GO and KEGG entries that are significantly enriched compared to the in-house genome background. Pathway enrichment analysis of DEGs was performed using the KEGG Automatic Annotation Server (KAAS).^2^

### Weighted co-expression network analysis and hub genes digging

Read counts of the whole transcript from all the 12 samples were subjected to R package WGCNA (v3.2.2) to construct gene co-expression networks. All samples were initially clustered to analyze the sample height. Following the application of the scale-free topology criterion, a soft threshold of 20 was chosen. Based on the topological overlap-based dissimilarity measure, unigenes were first hierarchically clustered, and the gene dendrogram was used for module detection by the dynamic tree cut method with mergeCutHeight = 0.25 and minModuleSize = 50. We calculated the correlation coefficients between the module eigengene (ME) and 26 flavonoid contents. In the WGCNA, gene connectivity was based on the edge weight (ranging from 0 to 1) determined by the topology overlap measure, which reflects the strength of the communication between genes. The weights across all edges of a node were summed and used to define the level of connectivity by Cytoscape plug-in CytoHubba, and flavonoids-related genes in a ME with high connectivity were considered as hub genes. Illustration of genes connectivity within a specific module was performed in Cytoscape 3.9.0.

### Whole-transcriptional identification of *AsOMT* and *UDP-glucuronosyltransferase* gene family

First, known plant OMT (Xie et al., 2022) or UGT78D2 protein sequences were subjected to the BLASTP process against the whole transcripts to find possible homologs. Second, an HMMER search for conserved domain structure of PF00891, S-adenosyl-L-methionine (SAM)-dependent methyltransferase, was conducted against the sandrice protein database on a local server. Union set of the two results above was then submitted to the NCBI web CD-search tool^3^ and manually checked to exclude sequences with incomplete conserved domain, resulting in a protein set of candidates AsOMT. For phylogenetic analysis of inter-species OMT tree and intra-species AsOMT and AsUGT78D2 trees, multi-alignment of OMT (or UGT78D2) protein sequences was conducted by MEGA X built-in Muscle program, and a neighborhood-joining (NJ) tree was constructed by MEGA X. Visualization of the phylogenetic trees and gene structure were performed in the iTOL online server^4^ and GSDS 2.0 online server,^5^ respectively; conserved motifs were found by the help of MEME suite^6^ while expression profile heatmaps, conserved domains, and conserved motifs were visualized by TBtools.

## Results

### Multiple environmental variables affect flavonoid accumulation in sandrice

A total of 26 types of flavonoids in all the 12 tested samples by flavonoids-targeted metabolomics were identified in our latest research, whereby isorhamnetin, isoquercitrin, quercetin, tricin, isorhamnetin-3G, and rutin were the top six flavonoids with the highest contents (average content ≥ 100 μg/g, Figure 1B; Zhou et al., 2021a). Various environmental factors co-shaped the unique original habitats. Thus, multivariance stepwise regression provides more robust evidence other than univariate linear regression and was conducted to take a closer aspect of the combined relationship between the 22 environmental climatic factors in the original habitats of the six ecotypes and 14 highly accumulated flavonoids in sandrice (Table 1). In general, the amounts of most flavonoids had a positive correlation with temperature and UVB, whereas negatively affected by precipitation and sunshine duration (SD). Among all the regression equations, resolution (adjusted *R*^2^) of rutin (0.736), hispidulin (0.672), quercetin (0.602), and tricin (0.601) were of the highest value with a *p*-value of 0, indicating the four equations provided better explanations to the relationship between corresponding flavonoid contents and environmental factors. The content of rutin was negatively affected by ALT (altitude) and *T*min (the average minimum temperature in June/August); hispidulin accumulation had a positive correlation with aat10 (accumulated temperature, ≥ 10°C) and *T*min, while was negatively affected by PWM (Precipitation of Wettest Month), MTWEQ (Mean Temperature of Wettest Quarter), and PRE (average precipitation in June/August). Equations in quercetin and tricin were found quite similar. These two flavonoids were both positively affected by PS (precipitation seasonality) and MTWEQ, whereas had negative correlations with SD and TAR (Temperature Annual Range), respectively. Isorhamnetin was negatively affected by PWM, while isoquercitrin had a positive correlation with aat10 and UVB, and a negative correlation with *T*max (the average maximum temperature in June/August), respectively. Though with rather low resolution (0.297), UVB positively affected the accumulation of isorhamnetin-3-glycoside in sandrice, which was in accordance with previous research in peach fruit (Xie et al., 2022).

**TABLE 1:** Multivariance stepwise regression equation of flavonoid contents and environmental factors.

Compounds	Adjusted *R*^2^	Equation of regression	*P*-value	*F*
Total flavonoids	0.116	Y = 2146.964–49.847PWEQ	0.011	7.035
Isorhamnetin	0.205	Y = 896.279–3.057PWM	0.001	12.827
Isoquercitrin	0.399	Y = –1120.472 + 0.024aat10+869.483UVB–26.172Tmax	0.000	9.530
Quercetin	0.602	Y = 2719.683+7.298PS–0.702SD+33.444MTWEQ–42.609TAR	0.000	18.379
Tricin	0.601	Y = 2470.176+6.726PS–0.629SD+31.760MTWEQ–40.106TAR	0.000	18.356
Isorhamnetin3G	0.297	Y = –681.872+479.657UVB	0.000	20.471
Rutin	0.736	Y = 802.331–0.168ALT-31.129Tmin	0.000	65.070
Isovitexin	NA	NA	NA	NA
Calycosin7G	0.242	Y = –127.505+1.594PS	0.000	15.646
Astragalin	0.115	Y = 35.448–0.136PWM	0.011	6.961
Hesperidin	0.217	Y = –4.412+0.005Arid	0.001	13.749
Hispidulin	0.674	Y = 38.108–0.072PWM+0.001aat10–2.5MTWEQ-0.129PRE+1.336Tmin	0.000	20
Luteoloside	0.132	Y = 12.820–0.052PWM	0.007	8.008
Kaempferol	0.396	Y = 33.452+1.997MMTR–0.019SD	0.000	16.066
Lonicerin	0.186	Y = 4.669–0.026PS	0.001	11.481

Flavonoids with an average amount of more than 1 μg/g were taken into consideration. The four equations with resolution (R^2^) > 0.5 were presented in bold. PWEQ, Precipitation of Wettest Quarter; PWM, Precipitation of Wettest Month; aat10, accumulated temperature, ≥ 10°C; UVB, UVB radiation; Tmax/min, the average maximum/minimum temperature of June or August; PS, Precipitation Seasonality (Coefficient of Variation); SD, sunshine duration; MTWEQ, Mean Temperature of Wettest Quarter; TAR, Temperature Annual Range; Alt, Altitude; Arid, aridity; PRE, average precipitation of June or August; MMTR, Mean Monthly Temperature Range.

### Biofunction prediction of transcripts and flavonoid biosynthesis-related differentially expressed genes identification

Paired-end RNA sequencing generated 8.67 Gb of raw data. After filtering, 8.63 Gb clean data with an average Q30 value of 91.06 were mapped to the sandrice reference genome (unpublished data), and the average mapping ratio of the 12 samples was about 94%. A total of 69,323 unigenes were generated by the HISAT2-Stringtie pipeline. The mean length of unigenes was 1,292 bp, with N50 of 9,388 bp and GC content of 38%, respectively. The statistics of length distribution showed that 22,917 unigenes ranged from 1,000 bp to 2,000 bp, accounting for 33.06% of the total sequences, while 16,531 unigenes were 2,000–3,000 bp, accounting for 23.85% of the total sequences (Supplementary Figure 1A). Annotation of all unigenes generated 38,624, 50,716, 44,967, 55,947, 57,655, 52,039 significant hits against GO, InterPro, SwissProt, Nr, TrEMBL, and TAIR databases, respectively (Supplementary Figure 1B). A total of 7,840 uncharacterized unigenes were designated as novel genes in sandrice.

**FIGURE 2:**
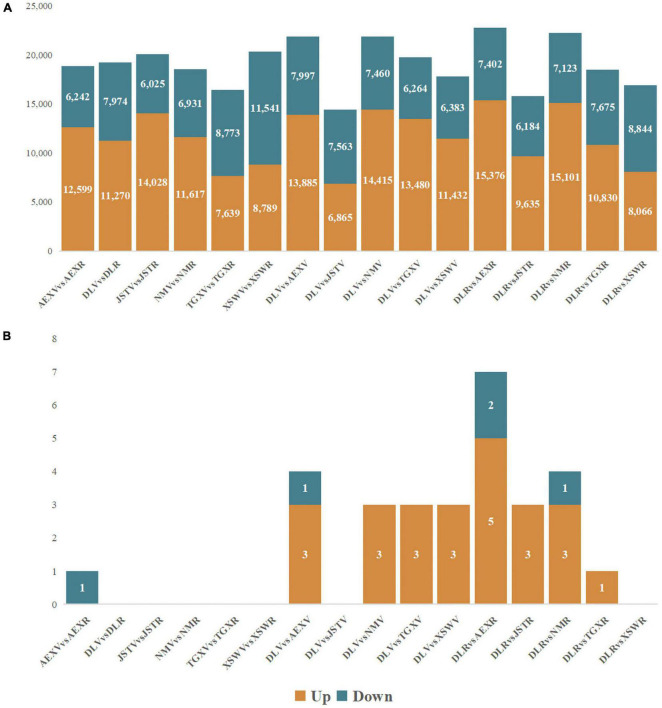
Pairwise comparison of DEG **(A)** and DAF **(B)**. Up- and downregulated genes or flavonoids are illustrated in orange and turquoise, respectively.

To clarify possible changes during different growth periods and variant gene background of sandrice on the transcriptional level, DEGs were identified among all the six ecotypes across the developmental stage. In general, more upregulated DEGs were identified in comparison between vegetative stage (V) and reproductive stage (R) than downregulated DEGs among most ecotypes, except for TGX and XSW (Figure 2A). Venn diagram of DEG_VvsR_ showed that all ecotypes shared 1,834 common DEG, representing the core transcriptional changes between vegetative and reproductive stages (Supplementary Figure 2A). Comparative KEGG enrichment of DEG_VvsR_ (Supplementary Figure 2C) unveiled that the six ecotypes have experienced the same transcriptional changes in “Starch and sucrose metabolism,” “Glycerophospholipid metabolism,” “GPI-anchor biosynthesis,” and “Terpenoid backbone biosynthesis.” However, flavonoid biosynthesis was not one of the characteristic differences between vegetative growth and reproductive growth in sandrice, which was in accordance with metabolomic analysis (Figure 2B).

Due to its lowest total flavonoid contents among all the ecotypes, we chose ecotype DL as CK to perform DEG identification among the other five ecotypes in the two developmental stages. In the vegetative stage, except for DLVvsJSTV, all the other comparisons had nearly twofold of upregulated DEGs than downregulated ones. However, in the reproductive stage, the comparison of DLRvsAEXR had the most numerous of DEG, indicating these two ecotypes were of great discrepancy in the reproductive stage (Figure 2A). As shown in Supplementary Figures 2B,D, the majority of DEG was shared among the five comparation in each of the developmental stages, and mainly distributed in KEGG terms “Glycerophospholipid metabolism,” “Phosphatidylinositol signaling system,” “GPI-anchor biosynthesis,” “Terpenoid backbone biosynthesis,” and “Ether lipid metabolism” (Supplementary Figure 2C). Flavonoid biosynthesis-related DEG was screened out based on their KEGG Orthology (ko00940, phenylpropanoid biosynthesis, and ko00941, flavonoid biosynthesis) in the enrichment result. A total of 24 flavonoids-related DEGs were found, of which five genes, *peroxidase* (*POD*), *4CL*, *COMT*, and *glycosyl hydrolase family protein* (*GHF*), were the most abundant flavonoids-related genes, the copy numbers of eight genes, *CHS*, *flavanone 3-dioxygenase* (*F3H*), *CHI*, *glycosyltransferase* (*GTF*), *Cytochrome P450 98A2* (*CYP98A2*), *feruloyl-CoA 6-hydroxylase* (*F6H*), *CCoAOMT*, and *flavanol synthase* (*FLS*), were of moderate quantity, while the rest 11 genes were scarcely found differentially expressed (Supplementary Figure 3). On the other hand, Members in one gene family have diverse expression patterns, for example, three copies of CCoAOMT-coding genes, *AsqAEX008163*, *AsqAEX019070*, and *AsqAEX013633*, were highly expressed in XSWR, NMV, and AEXV, respectively. In general, more highly expressed flavonoid-related genes were found in AEX and NM in the two developmental stages than in other samples, while only a few genes expressed in DL and TGX (Supplementary Figure 4), which could be due to the different stress intensity impacted on the variant genetic background of these ecotypes in the common garden trial.

### Weighted co-expression network analysis unveiled hub genes in key flavonoids regulation in sandrice

**FIGURE 3:**
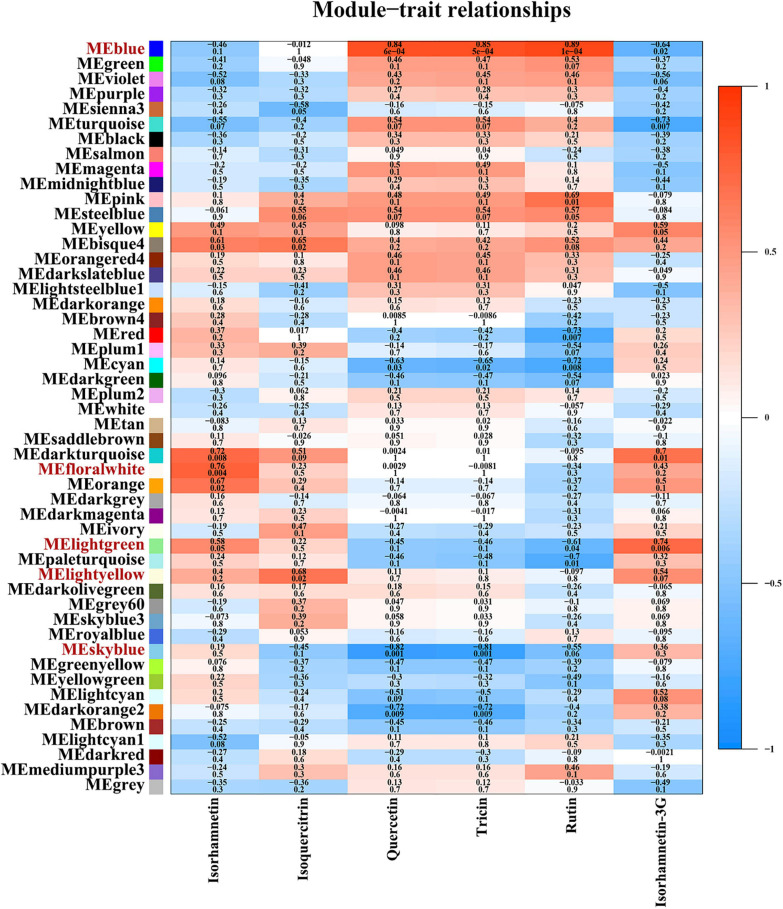
Module–trait relationships, partial. This figure shows relationships between 50 module eigengenes and the top six flavonoids. The complete module-trait relationships involving all the 26 detected flavonoids was provided in Supplementary Figure 6. The correlation and *p*-value of corresponding flavonoids and gene modules are shown as numbers up and down in each cell, respectively. The color scale on the right side represents the module–trait correlation from −1 (blue) to 1 (red).

WCGNA integrating transcriptomics and metabolomics was conducted to find the regulation pattern of flavonoids in sandrice. A total of 46,391 (66.9%) unigenes (out of 69,323; after filtering the ones with low expression) were subjected to WGCNA for the characterization of gene networks in flavonoid biosynthesis. Based on their expression profiles, the tested 12 samples were clearly divided in PCA, whereby AEX and NM, the two low-altitude ecotypes, were separated from the other samples along dim1; while the vegetative stage and reproductive stage of each ecotype were divided along dim2 (Supplementary Figure 5A). A scale-free topology model with a soft threshold of 20 (Supplementary Figures 5B,C) was used, resulting in 50 module eigengenes (ME) according to gene co-expression patterns (Supplementary Figures 5D,E). Then, an investigation of correlations was conducted between the 50 co-expression modules and the top six flavonoids in sandrice (Figure 3). We identified four MEs that were significantly (*p* < 0.05) correlated with the top six flavonoids in sandrice, which were MEblue with highly positive coefficients associated with quercetin (0.84), tricin (0.85), and rutin (0.89); MEfloralwhite, with isorhamnetin (0.76); MElightgreen, with isorhamnetin-3-glycoside (0.74); and MElightyellow, with isoquercitrin (0.68), respectively. Additionally, quercetin and tricin were significantly negatively correlated with MEskyblue (−0.82 and −0.81, respectively). We also found some high correlations in the module–traits relationship, such as biochanin A (MEpink, 0.96), isovitexin (MEdarkred, 0.96), hesperidin (MEdarkgray, 0.99), naringenin, and eriodictyol (MElightsteelblue1, 0.94 and 0.93, respectively), providing insights into the utilization of the corresponding compounds in the future (Supplementary Figure 6).

**FIGURE 4:**
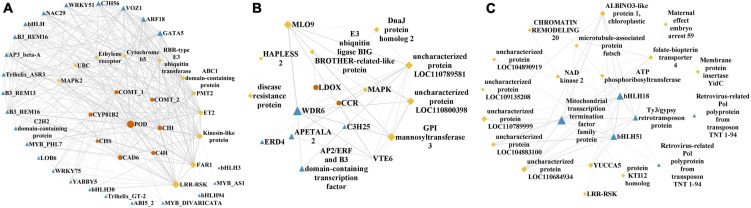
Hub genes identified in flavonoids-correlated modules. Hub genes in **(A)** MEblue, **(B)** MEskyblue, and **(C)** MEfloralwhite, respectively. Flavonoid biosynthesis genes, transcription factors or regulators, and other high connectivity genes are shown as orange dots, blue triangle, and yellow diamonds, respectively. Shape size indicated connectivity, while line width indicated the weight of edges.

KEGG enrichment of the five modules with high correlations with the top flavonoids in sandrice mentioned above, i.e., MEblue, MEskyblue, MElightgreen, MElightyellow, and MEfloralwhite, was performed to clarify their potent biofunction (Supplementary Table 3). Next, hub genes in MEblue, MEskyblue, and MEfloralwhite were screened out based on their annotation and connectivity in the corresponding module (Figure 4). MEblue had a highly positive correlation with quercetin, tricin, rutin, and naringin, and contained eight flavonoid synthases, including POD, CHI, CHS, C4H, cinnamyl alcohol dehydrogenase (CAD), cytochrome P450 81B2 (CYP81B2), and two COMTs. We found a lot of TFs in this module, such as four bHLHs, three MYBs, three B3s, two WRKYs, etc. Interestingly, FAR-RED IMPAIRED RESPONSE 1 (FAR1) was also found in this module, indicating that quercetin, tricin, and rutin might be involved in plants’ light signaling transduction and photomorphogenesis (Figure 4A; Ma and Li, 2018). MEskyblue was in negative correlation with quercetin and tricin. A member of the WD-domain repeat (WDR) TF family, WDR6, was screened out in MEskyblue (Figure 4B). WDR family was documented as a component of the MYB-bHLH-WDR (MBW) complex that regulates the biosynthesis of flavonoids, especially anthocyanin, and leucoanthocyanidin dioxygenase (LDOX) is one of its targets (Xu et al., 2015). Besides, other four TFs, including APETALA2, AP2/ERF, C3H25, and early responsive to dehydration stress 4 (ERD4), were found negatively regulated the accumulation of quercetin and tricin (Figure 4B). In MEfloralwhite, the module with a positive correlation with isorhamnetin, two members in the *bHLH* TF family, *bHLH 18* and *bHLH 51*, were identified as hub genes, indicating the two TFs might play key roles in the regulation of isorhamnetin biosynthesis (Figure 4C). A few uncharacterized genes and three transposons, two transposons TNT 1–94 and one member in Ty3/Gypsy family, were involved in the regulation of isorhamnetin, whose function requires further study (Figure 4C). In MElightgreen and MElightyellow, with a high correlation with isoquercitrin and isorhamnetin-3-glycoside, respectively, no flavonoids-related gene was detected. Details of hub genes in each module are listed in Supplementary Table 4.

### Identification of putative isorhamnetin and isorhamnetin-3-glycoside biosynthesis in sandrice

**FIGURE 5:**
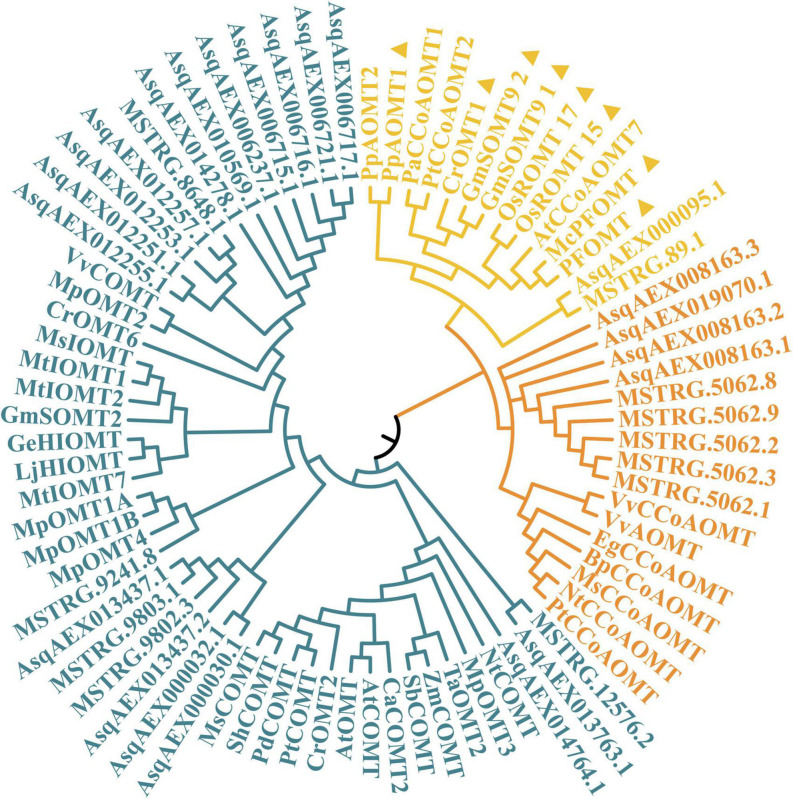
Phylogenetic relationship of 33 candidate AsOMT and 45 known OMTs in other plants. CCoAOMT subfamily, PFOMT subclade, and COMT subfamily are colored in orange, yellow, and blue, respectively. Sequences marked with triangles are the reported isorhamnetin biosynthesis genes.

**FIGURE 6:**
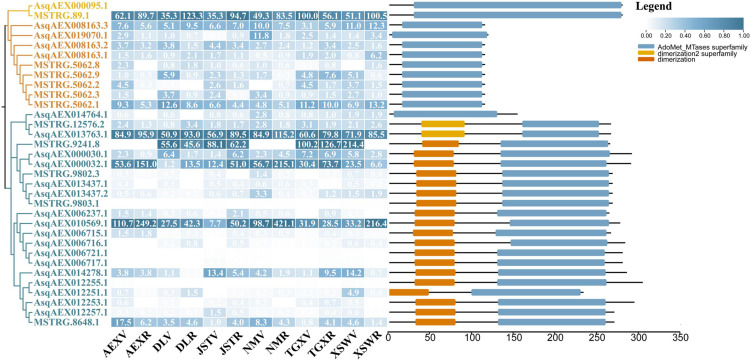
*AsOMT* gene family identification. From left to right, the three columns showed the phylogenetic tree, heatmap of expression profiles (transcripts per million, TPM) in each of the 12 samples, and conserved domain visualization of the 33 candidates AsOMT, respectively. In the phylogenetic tree, the *AsPFOMT* subclade, *AsCCoAOMT* subfamily, and *AsCOMT* subfamily are shaded in yellow, orange, and blue, respectively. Numbers in each cell of the expression profile heatmap are genes’ original TPM values. Key conserved domains are shown as different colored bars, according to the actual length of peptides.

Members of the *OMT* gene family were reported in previous studies to code the O-methyltransferases that catalyze methylation on quercetin at the 3′-OH to form isorhamnetin. As shown in Figure 5, a total of 33 sandrice candidate OMTs were identified based on the whole transcripts, including 11 members in the CCoAOMT subfamily containing two members in the PFOMT subclade, and 22 members in the COMT subfamily. OMTs specifically catalyze the biosynthesis of isorhamnetin in other plants, such as GmSOMT9, OsROMT, and McPFOMT, etc., were all located in a subclade PFOMT in the CCoAOMT subfamily (Xie et al., 2022), and clustered with sandrice transcripts *MSTRG89.1* and *AsqAEX000095.1*. Of note, AsOMT shared relatively low similarity with OMTs in other species and were clustered into solely subclades, indicating OMT in sandrice had experienced early differentiation and possibly subsequent gene duplication events. The intra-species AsOMT phylogenetic tree was divided into two subfamilies, CCoAOMT and COMT, in accordance with the inter-species tree (Figure 6). Members in the CCoAOMT subfamily embraced the conserved domain AdoMet_MTases_superfamily, and the COMT subfamily contained a dimerized domain and an AdoMet_MTases domain. In the AsPFOMT subclade, *AsqAEX000095.1* had low expression in the 12 samples. Thus, *MSTRG89.1* might be the candidate PFOMT-coding gene in isorhamnetin biosynthesis in sandrice.

On the other hand, isorhamnetin-3-glycoside is formed by glycosylation on isorhamnetin at 3-OH. Gene *UGT78D2* was speculated to encode glycosyltransferases and act on isorhamnetin to generate isorhamnetin-3-glycoside. To figure out the isorhamnetin-3-glycoside biosynthesis route in sandrice, a total of 14 candidates sandrice *UGT78D2* genes were identified, among which 12 genes were expressed effectively in the 12 samples (Supplementary Figure 7). Some of them present ecotype- and/or developmental stage-specific expression modes, such as *AsqAEX021169.1* and *AsqAEX021169.2*. These two genes had almost identical gene structures and conserved motifs but expressed complementarily in the 12 samples. *MSTRG.19033.1* expressed specifically in low-altitude ecotypes, AEX and NM, indicating the accumulation of isorhamnetin-3-glycoside might be involved in stress resistance (Supplementary Figure 7).

### The molecular basis of flavonoid biosynthesis in sandrice

OPLS-DA analysis between all the six ecotypes across the two developmental stages unveiled that there was no obvious change in flavonoid contents during sandrice growth; whereas, the reproductive stage of DL (DLR) and AEX (AEXR) had the most discrepancy in flavonoid accumulation (Figure 2B), of which contents of total flavonoids, quercetin, tricin, rutin, and isoquercitrin were higher in AEXR than DLR, while accumulation of isorhamnetin and isorhamnetin-3-glycoside were higher in DLR than in AEXR, significantly (Table 2 and Supplementary Figure 8).

**TABLE 2:** DAF (DLRvsAEXR) identified by OPLS-DA.

Compounds	Log2FC	VIP	*P*-value
Total flavonoids	0.40	2.56	0.02
Isorhamnetin	–0.68	2.22	0.02
Quercetin	2.39	2.15	0.00
Tricin	2.46	2.04	0.00
Rutin	2.37	1.79	0.00
Isoquercitrin	1.15	1.37	0.00
Isorhamnetin3G	–1.09	1.06	0.00

VIP, Variable important in projection.

To clarify the molecular basis underlying metabolomic differences between AEXR and DLR, biofunction prediction of DEG_DLRvsAEXR_ was conducted based on transcriptomics. Compared to DLR, there were 15,376 and 7,402 up- and downregulated DEGs identified in AEXR, respectively (Figure 2A). GO enrichment showed that the top five enriched terms were cytoplasm (GO:0005737), carbohydrate binding (GO:0005529), ABC-type transporter activity (GO:0140359), RNA processing (GO:0006396), and kinase activity (GO:0016301; Supplementary Figure 9A), while the KEGG enrichment analysis categorized these genes mainly into Biosynthesis of secondary metabolites (ko01110), carbon metabolism (ko01200), starch and sucrose metabolism (ko00500), biosynthesis of amino acids (ko01230), phenylpropanoid biosynthesis (ko00940) in class metabolism, spliceosome (ko03040) in class Information Processing, and plant hormone signal transduction (ko04075) in class Environmental Information Processing (Supplementary Figure 9B).

**FIGURE 7:**
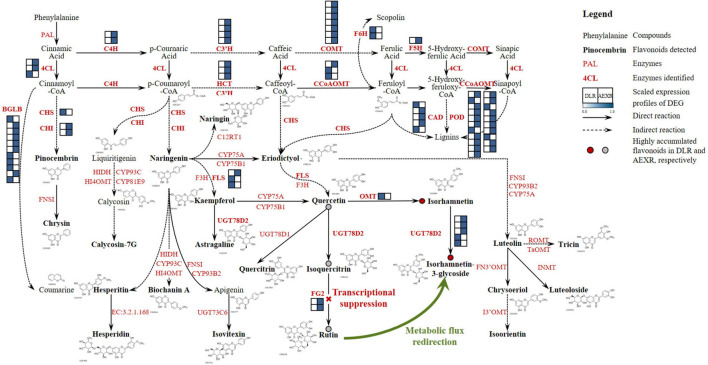
Flavonoid biosynthesis pathway in sandrice. Detected flavonoids and DEG_DLRvsAEXR_ in this research are shown in bold fonts, black and red, respectively. The structural formula of the corresponding compound is also presented. DAF between AEXR and DLR are shown as dots, whereby red and gray dots indicate up- and downregulated flavonoids in DLR than in AEXR, respectively. The red cross and green curved arrow indicate the possible mechanism of trade-off between rutin and isorhamnetin biosynthesis. 4CL, 4-coumarate–CoA ligase; BGLB, Beta-glucosidase; C3′H, 5-O-(4-coumaroyl)-D-quinate 3′-monooxygenase; C4H, Trans-cinnamate 4-monooxygenase; CAD, Cinnamyl alcohol dehydrogenase; CCoAOMT, Caffeoyl-CoA O-methyltransferase; CHI, Chalcone-flavanone isomerase; CHS, Chalcone synthase; COMT, Caffeic acid 3-O-methyltransferase; CYP75A, Flavonoid 3′,5′-hydroxylase; CYP75B1, Flavonoid 3′-monooxygenase; CYP93B2, Flavone synthase II; CYP93C, 2-hydroxyisoflavanone synthase; CYP98A2, Cytochrome P450 98A2; F3H, Flavanone 3-dioxygenase; F3′H, Flavonoid 3′hydroxylase; F5H, Cytochrome P450 84A1; F6H, Feruloyl-CoA 6-hydroxylase; FG2, Flavonol-3-O-glucoside L-rhamnosyltransferase; FLS, Flavanol synthase; FN3′OMT, Flavone 3′-O-methyltransferase; FNSI, Flavone synthase I; GHF, Glycosyl hydrolase family protein; GTF, Glycosyltransferase; HCT, Shikimate O-hydroxycinnamoyltransferase; HI4OMT, 2,7,4′-trihydroxyisoflavanone 4′-O-methyltransferase/isoflavone 4′-O-methyltransferase; HIDH, 2-hydroxyisoflavanone dehydratase; I3′OMT, Isoorientin 3′-O-methyltransferase; INMT, Amine N-methyltransferase OMT, SAM-dependent O-methyltransferase; PAL, Phenylalanine ammonia-lyase; POD, Peroxidase; ROMT, Tricin synthase; TaOMT, Tricetin 3′,4′,5′-O-trimethyltransferase; UGT73C6, Flavonol-3-O-L-rhamnoside-7-O-glucosyltransferase; UGT78D1, Flavonol-3-O -rhamnosyltransferase; UGT78D2, Flavonol-3-O-glycosyltransferase; EC:3.2.1.168, Hesperidin 6-O-alpha-L-rhamnosyl-beta-D-glucosidase.

A flavonoid biosynthesis network in sandrice containing the detected 23 compounds was then constructed (Figure 7). The pathway of sandrice flavonoid biosynthesis starts with the transformation of phenylalanine to p-coumaroyl-CoA. The two main outlets of the GPP, lignin biosynthesis, and flavonoid biosynthesis, were shunted here, *via* generating naringenin and a series of CoA, respectively (Dong and Lin, 2021). The majority of the flavonoids-related DEG_DLRvsAEXR_ (51 out of 85) was identified in the lignin biosynthesis pathway, including three copies of *4CL*, two *C4H*s, four *Cytochrome P450 98A2*s (*C3′H*), three *shikimate O-hydroxycinnamoyltransferase*s (*HCT*s), seven *COMT*s, three *CCoAOMT*s, two *F6H*s, one *cytochrome P450 84A1* (*F5H*), five *CAD*s, and 21 *POD*s. Most of them had higher expression in AEXR than DLR, indicating lignin biosynthesis was activated in ecotype AEX, and possibly resulted from stresses (Meng et al., 2022). On the other hand, naringenin is the intermediate product of biosynthesis pathways of different classes of flavonoids (Zhao et al., 2020), from which 21 out of all the 23 detected flavonoids, except pinocembrin and chrysin, originated. However, only a few enzyme-coding genes were found in the flavonoid biosynthesis pathway in sandrice, i.e., two copies of *CHI*, one *CHS*, three *FLS*s, two *flavonol-3-O-glucoside L-rhamnosyltransferases* (*FG2*), one putative *OMT*, and five putative *UGT78D2*s. Lignin biosynthesis is one of the most well-studied pathways and is conserved in higher plants to a large extent, while clarifying flavonoid biosynthesis genes in small species required further endeavors, which might partially explain the reason why more genes were identified in lignin biosynthesis than flavonoids biosynthesis in sandrice. Quercetin is an important intermediate and serves as a tee-valve in sandrice flavonoid biosynthesis, from which three biosynthesis routes generate: quercetin-quercitrin; quercetin-isoquercitrin-rutin; and quercetin-isorhamnetin-isorhamnetin-3-glycoside (Figure 7). UGT78D1, UGT78D2, and PFOMT, respectively, catalyze the formation of quercitrin, isoquercitrin, and isorhamnetin. UGT78D2 also acts on isorhamnetin to form isorhamnetin-3-glycoside, while FG2 acts on isoquercitrin to form rutin. Interestingly, according to OPLS-DA, the contents of quercetin, isoquercitrin, and rutin were significantly lower in DLR, while isorhamnetin and isorhamnetin-3-glycoside accumulated higher in DLR (Table 2), indicating metabolic flux was somehow redirected to isorhamnetin generating rather than isoquercitrin generating in ecotype DL (Figure 7).

## Discussion

### Flavonoid accumulation in sandrice resulted more from stress-responding than other local adaptation

Flavonoid accumulation can be induced by multiple stresses and is regarded as the consequence of local adaptation in some species. For instance, highland *Zay maize* often accumulates flavonoids in its leaves for coping with the UV-B radiation (Casati and Walbot, 2005). Based on two common garden trials, three flavonoid biosynthesis-related genes, *anthocyanidin synthase* (*ANS*), *anthocyanidin reductase* (*ANR*), and *flavonoid 3’hydroxylase* (*F3*′*H*) were found outliers among seven natural population of *Pinus yunnanensis* (Sun et al., 2020). In Populus, multiple flavonoid-related genes were subjected to environment selection pressure and the region-specific allele played an important role in local adaptive evolution (Lu et al., 2021). Moreover, in our previous study, *CCoAOMT*, *F3*′*H*, and *HCT* were under diversifying selection along elevation gradients, indicating flavonoid accumulation was involved in sandrice high-altitude adaptation (data unpublished). Nevertheless, neither selective signal nor gene structure variance in flavonoid-related genes was detected in the present research (data not displayed). Furthermore, in multivariance stepwise regression analysis, most flavonoids had a low resolution in correlation with environmental factors in their original habitats (Table 1), indicating that local adaptation was not enough to explain the diversity of flavonoid accumulation among sandrice ecotypes. The common garden trial is a method of detecting local adaptation as well as studying phenotypic plasticity, which provides an equivalent field condition that eliminated environmental heterogeneity in ecotypes’ original habitats, while simultaneously creating new stresses (Dorman et al., 2009). Hence, the phenotype observed in a common garden (flavonoid accumulation in this research) was a consequence of genetic background that was generated from local adaptation (G), response to environmental cues (E), and their combined effects (G × E; Del Valle et al., 2018; Zhou et al., 2021a). WW (common garden location) was of mid-value among a series of environmental factors in the six original habitats. The diversity in expression profiles of flavonoid-related DEGs among different ecotypes (Supplementary Figure 4) indicated that low-land ecotypes (AEX and NM) were subjected to intense stress, such as dehydration and intense UVB radiation, while highland ecotypes, e.g., DL and TGX, were in stress relieve or stress release. In a word, flavonoid accumulation in sandrice is more like a consequence of response to environmental factors other than local adaptation to *in situ* habitats. Unfortunately, we were unable to quantify the influences of genetic backgrounds, environmental factors, and the much trickier factor, phenotypic plasticity. In this regard, genetic–environment interactions (G × E) analysis with strict variable control will be our future research direction.

### Flavonoid biosynthesis pathway in sandrice

Flavonoids obtain their structural biodiversity from a series of enzymatic substitution reactions, whereby methylation and glycosylation are two prevalent processes (Shelton et al., 2012; Wang S. et al., 2019). In the flavonoid biosynthesis pathway, quercetin serves as an important intermediate and generates isorhamnetin, quercitrin, and isoquercitrin. PFOMT catalyzed methyl transferring on quercetin 3′-OH to form isorhamnetin. It is reported that isorhamnetin rarely significantly accumulates in plants (Huang et al., 2004). However, our latest research showed isorhamnetin was highly enriched in sandrice multiple ecotypes despite different genetic backgrounds, indicating this compound is of great value to sandrice growth and development. In sandrice, homologs of *PFOMT* had either low expression or low correlation with isorhamnetin contents (Figure 6). This may be partially due to the complexity and reticulum of the flavonoid biosynthesis pathway. As an intermediate in the biosynthetic cascade of methylated flavonoids, isorhamnetin accumulation is mainly affected by its direct precursor and downstream compounds (quercetin and isorhamnetin-3-glycoside, respectively), and more importantly, the activity and catalytic efficiency of PFOMT. Therein, more experimental evidence, *in vivo* or *in vitro*, will help clarify the functions and regulatory modules of *AsPFOMT*.

Since flavonoids glycosylation results in an increase in stability (through the protection of reactive nucleophilic groups) and water solubility, natural flavonoids in plants are mostly found in the form of glycosides (Gachon et al., 2005). Two homologous enzymes in the *UGT78* gene family catalyze the first 3-O-glycosylation steps on flavonols, whereby UGT78D1 and UGT78D2 catalyzed rhamnosyl and glucosyl transferring on quercetin 3-OH, respectively (Le Roy et al., 2016), and UGT78D2 also acts on isorhamnetin to form isorhamnetin-3-glycoside (Figure 7). In this study, the *AsUGT78D2* gene family embraced 14 genes. Most enzymes in the flavonoid biosynthesis pathway are encoded by multi-genes families rather than a major gene (Vogt, 2010). Though we had little clue about the biological implications of the redundant function of members in a multi-gene family, it can be inferred as strategies in facing variant environment cues. Even though none of the *AsUGT78D2* family members had a significant positive correlation with isorhamnetin-3-glycoside content in sandrice, the 14 homologs were of diverse expression profiles (Supplementary Figure 7) and were considered to function redundantly and complementarily in isorhamnetin-3-glycoside biosynthesis, under the circumstance of adverse condition and different developmental stages.

### Trade-off between isorhamnetin and rutin might contribute to stress-responding in sandrice

Interestingly, the highland ecotype DL had relatively low contents of quercetin, isoquercitrin, and rutin, but was abundant in isorhamnetin and isorhamnetin-3-glycoside (Figure 1B), which was significant when compared to AEX (Table 2). Ecotype DL might redirect metabolic flux from route quercetin-isoquercitrin-rutin to route quercetin-isorhamnetin-isorhamnetin-3-glycoside by transcriptional suppression on *FG2* (Figure 7). DL locates at the Qinghai-Tibet Plateau, which is of the highest altitude (3,130 m) among the six tested ecotypes, with a combination of ecological factors of low precipitation, low temperature, long SD, and intense UVB radiation (Supplementary Table 1). Our previous studies depicted that quercetin was abundant in sandrice highland ecotype, and over-accumulated in lowland ecotype after transplanting into the middle-altitudinal common garden, indicating quercetin can respond to environmental factors involved in high-altitudinal habitats. The character of quercetin has been thoroughly studied. It is reported that the high efficiency in ROS elimination of quercetin rendered plants with prevalent protection against intense UVB radiation, heavy metal, osmotic stress, microbial infection, etc. Moreover, quercetin could induce phytohormone signaling pathways, including auxin (IAA) and abscisic acid (ABA), and regulate the activity of enzymatic and non-enzymatic antioxidants in a dose-dependent manner (Jańczak-Pienia̧żek et al., 2021; Singh et al., 2021). On the other hand, rutin was documented to be a plant defense compound against cold stress (Reimer et al., 2022), salinity (Lim et al., 2012), and accumulated after UVB radiation (Huang et al., 2016). Limited studies on isorhamnetin function in plants *in vivo* unveiled that isorhamnetin and isorhamnetin-3-glycoside could respond to UVB, while the latter was upregulated by fungus infection (Santin et al., 2018; Xie et al., 2022). According to research on the antioxidant property of quercetin and its derivates, quercetin is believed to have the highest potential, followed by isorhamnetin and isorhamnetin-3-glycoside, whereas in terms of lipid-peroxidation inhibition, isorhamnetin showed higher activities than quercetin (Singh et al., 2021). If isorhamnetin and its derivate can respond to stresses occurred in the common garden, then enrichment of isorhamnetin should have been observed in lowland ecotypes; If quercetin, isoquercitrin, and rutin are responsible for ROS elimination, then a similar decrease in biosynthesis of these three flavonoids should have been monitored in the other highland ecotype TGX. However, no such phenomenon was observed. The reason for the trade-off between rutin and isorhamnetin in sandrice ecotype DL remains unclear, but the unusual high accumulation of isorhamnetin and isorhamnetin-3-glycoside indicated that the ecotype might readjust the homeostasis between quercetin (isoquercitrin or rutin) and isorhamnetin (and isorhamnetin-3-glycoside) to cope with environmental changes.

## Conclusion

In this research, we conducted multivariance stepwise regression analysis and elucidated the main environmental factors, i.e., precipitation, temperature, UVB, and sunshine duration, that affect flavonoid accumulation in sandrice. Based on a common garden trial, an integrated analysis of transcriptomics and flavonoids-targeted metabolomics from six sandrice ecotypes in vegetative and reproductive stages provided valuable data in outlining the flavonoid biosynthesis pathway in sandrice. WGCNA and hub gene digging illustrated the molecular regulatory network underlying the biosynthesis of the characteristic flavonoids in sandrice, for instance, isorhamnetin, quercetin, rutin, isoquercitrin, tricin, and isorhamnetin-3-glycoside, whereby *bHLH* and *MYB* TF family might be the key transcriptional regulatory elements in flavonoid accumulation. Moreover, identification and characterization the of *AsPFOMT* gene family and *AsUGT78D2* gene family unveiled that members in the two families might serve as synthases of isorhamnetin and isorhamnetin-3-glycoside, respectively. Finally, the hypothesis that the trade-off between rutin and isorhamnetin in highland ecotype DL provided insights into sandrice stress-responding mechanisms. Although there are still many gaps to be filled, the present research provided preliminary but valuable data support in elucidating the molecular basis of sandrice flavonoid biosynthesis, paving the way to precise development of this valuable resource forage in the health industry.

## Data availability statement

The data presented in this study are deposited in the NCBI SRA repository, accession number: PRJNA853545.

## Author contributions

X-FM: conceptualization. TF, SZ, XYY, and YL: data curation. CQ and X-FM: funding acquisition and writing—review and editing. SZ: investigation. CQ, XY, and YC: methodology. TF and XF: software. XF, PZ, and LS: validation. TF and XYY: visualization. TF: writing—original draft. All authors listed have made a substantial, direct, and intellectual contribution to the work, and have read and agreed to the published version of the manuscript.

## Footnotes

1
https://www.omicshare.com/tools/

2
http://www.genome.jp/tools/kaas/

3
https://www.ncbi.nlm.nih.gov/Structure/cdd/wrpsb.cgi

4
https://itol.embl.de/

5
http://gsds.gao-lab.org/

6
https://meme-suite.org/meme/
