# Corrigendum: Integrated metabolomics and transcriptomics insights on flavonoid biosynthesis of a medicinal functional forage, *Agriophyllum squarrosum* (L.), based on a common garden trial covering six ecotypes

**DOI:** 10.3389/fpls.2022.1092707

**Published:** 2022-11-23

**Authors:** Tingzhou Fang, Shanshan Zhou, Chaoju Qian, Xia Yan, Xiaoyue Yin, Xingke Fan, Pengshu Zhao, Yuqiu Liao, Liang Shi, Yuxiao Chang, Xiao-Fei Ma

**Affiliations:** ^1^ Key Laboratory of Stress Physiology and Ecology in Cold and Arid Regions, Department of Ecology and Agriculture Research, Northwest Institute of Eco-Environment and Resources, Chinese Academy of Sciences, Lanzhou, China; ^2^ College of Resources and Environment, University of Chinese Academy of Sciences, Beijing, China; ^3^ Faculty of Environmental Science and Engineering, Shanxi Institute of Science and Technology, Jincheng, China; ^4^ Key Laboratory of Eco-Hydrology of Inland River Basin, Northwest Institute of Eco-Environment and Resources, Chinese Academy of Sciences, Lanzhou, China; ^5^ Marsgreen Biotech Jiangsu Co., Ltd., Haian, China; ^6^ Agricultural Genomics Institute at Shenzhen, Chinese Academy of Agricultural Sciences, Shenzhen, China

**Keywords:** *Agriophyllum squarrosum*, sandrice, flavonoids, transcriptome, metabolome, common garden, isorhamnetin-3-glycoside, isorhamnetin

In the published article, there was an error in affiliation 5. Instead of “Marsgreen Biotech Jiangsu Co., Ltd., Jiangyin, China”, it should be “Marsgreen Biotech Jiangsu Co., Ltd., Haian, China”.

There was an error regarding the affiliations for Tingzhou Fang, Pengshu Zhao, Yuqiu Liao, and Liang Shi. An affiliation was missed, “College of Resources and Environment, University of Chinese Academy of Sciences, Beijing, China”.

The authors apologize for these errors and state that this does not change the scientific conclusions of the article in any way. The original article has been updated.

## Publisher’s note

All claims expressed in this article are solely those of the authors and do not necessarily represent those of their affiliated organizations, or those of the publisher, the editors and the reviewers. Any product that may be evaluated in this article, or claim that may be made by its manufacturer, is not guaranteed or endorsed by the publisher.

