# Supplementary material for: Integrated metabolomics and transcriptomics insights on flavonoid biosynthesis of a medicinal functional forage, Agriophyllum squarrosum (L.), based on a common garden trial covering six ecotypes
Source: Front Plant Sci. 2022 Sep 20;13:985572. doi: 10.3389/fpls.2022.985572 (PMC9530573; doi:10.3389/fpls.2022.985572)
Supplement: Supplementary file 2 [file Data_Sheet_2.docx]

Supplementary Material

## Supplementary Figures

| 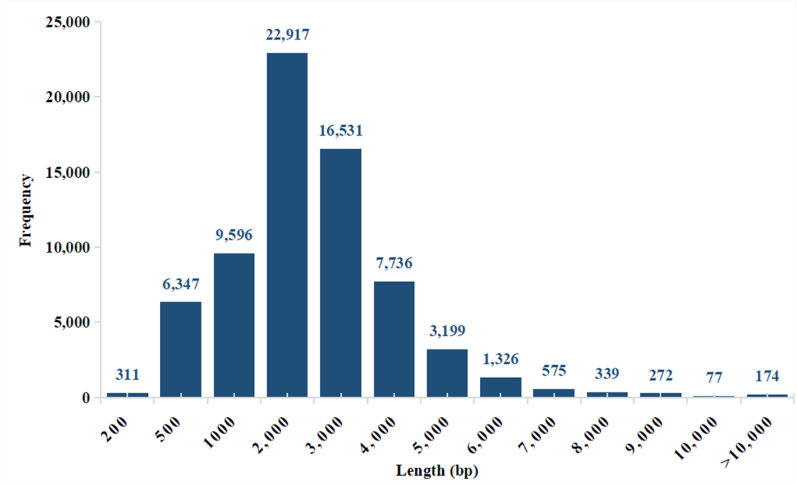 | 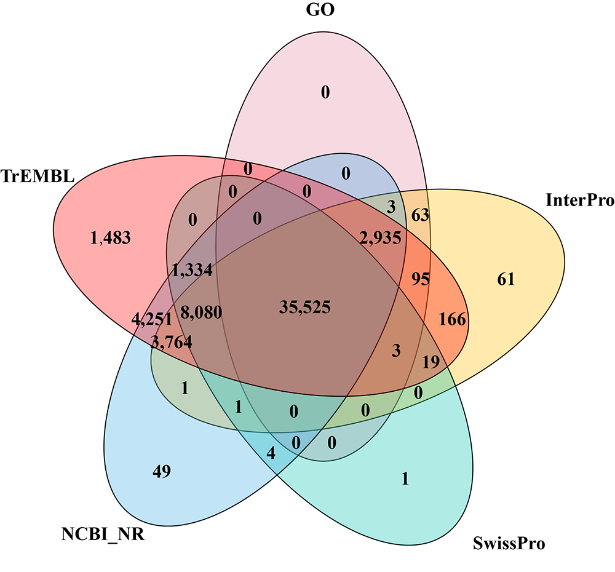 |
| --- | --- |
| (**A**) | (**B**) |

**Supplementary Figure 1.** Statistics of assembly and annotation of RNA-Seq. (**A**), Distribution of transcripts lengths; (**B**), Veen diagram of annotated gene numbers against the five databases.

| 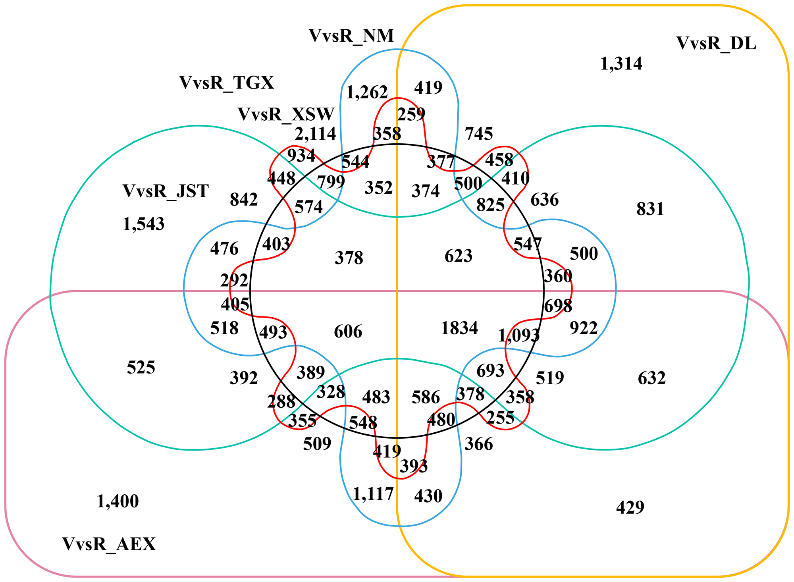 | 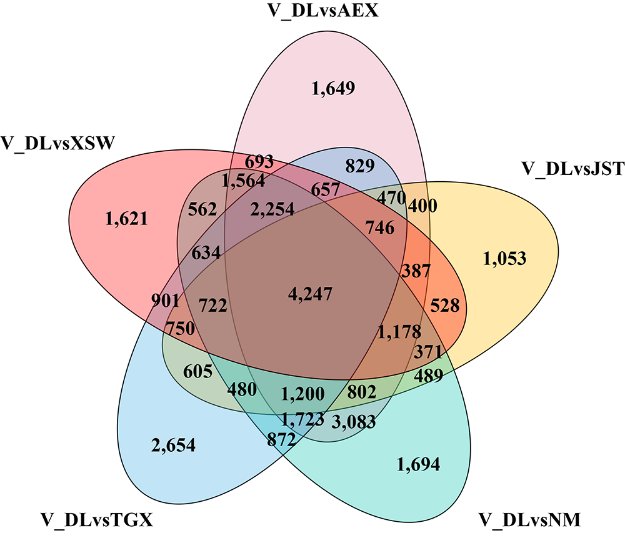 |
| --- | --- |
| (**A**) | (**B**) |
| 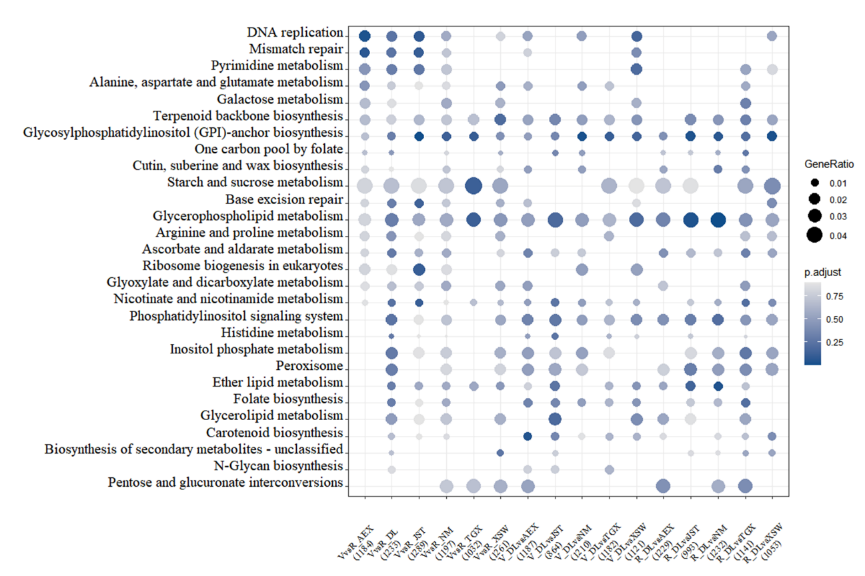 | 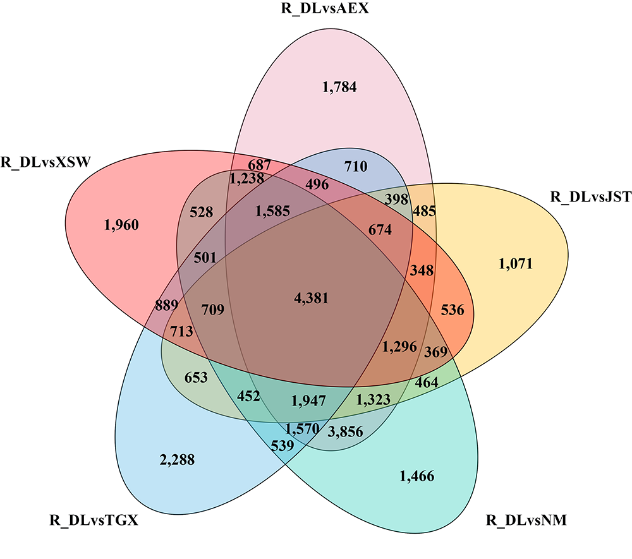 |
| (**C**) | (**D**) |

**Supplementary Figure 2.** Overlaps and KEGG enrichment of DEGs. (**A**), overlaps of comparison between different developmental stages within each ecotype; the other five ecotypes compared to DL in (**B**) vegetative stage and (**D**) reproductive stage; (**C**), KEGG enrichment of all the DEG comparison.

**
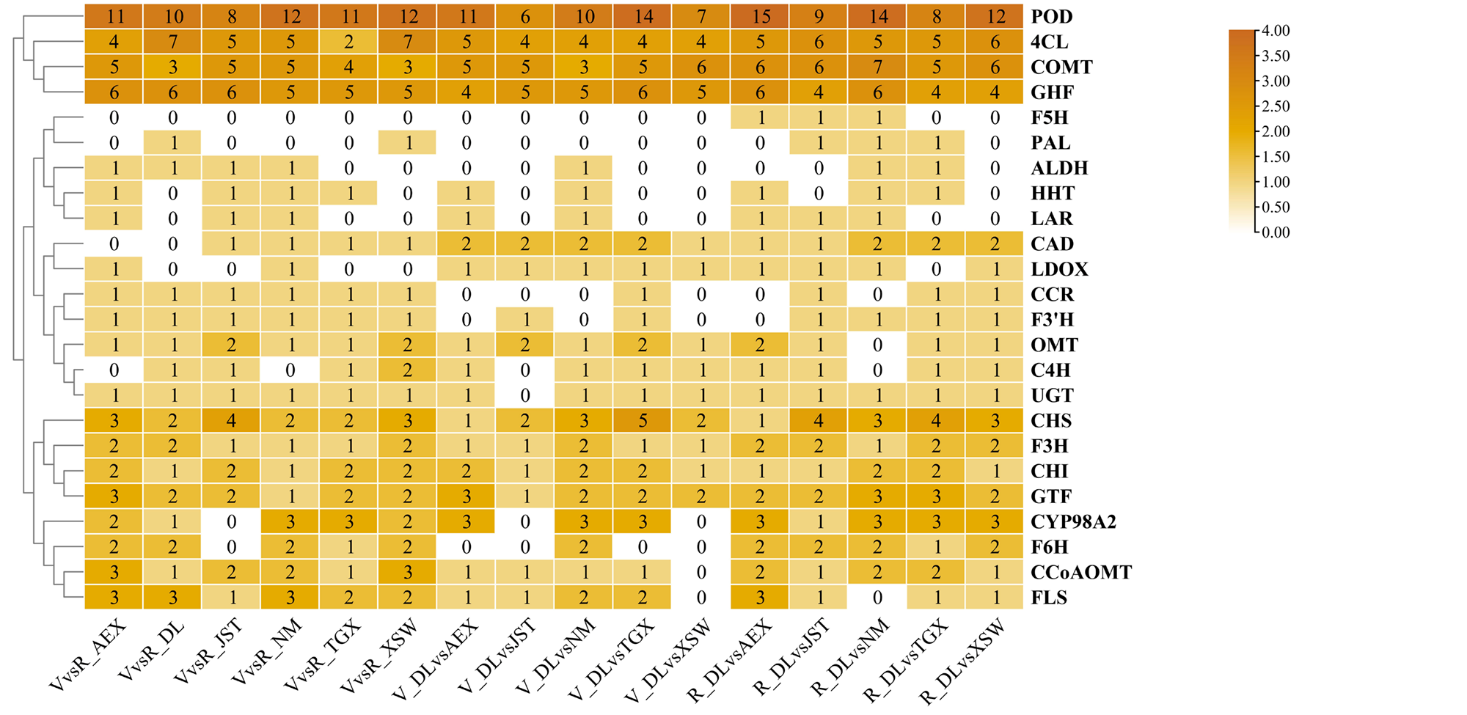
**

**Supplementary Figure 3.** Flavonoid biosynthesis-related DEGs identified in each comparison.

**
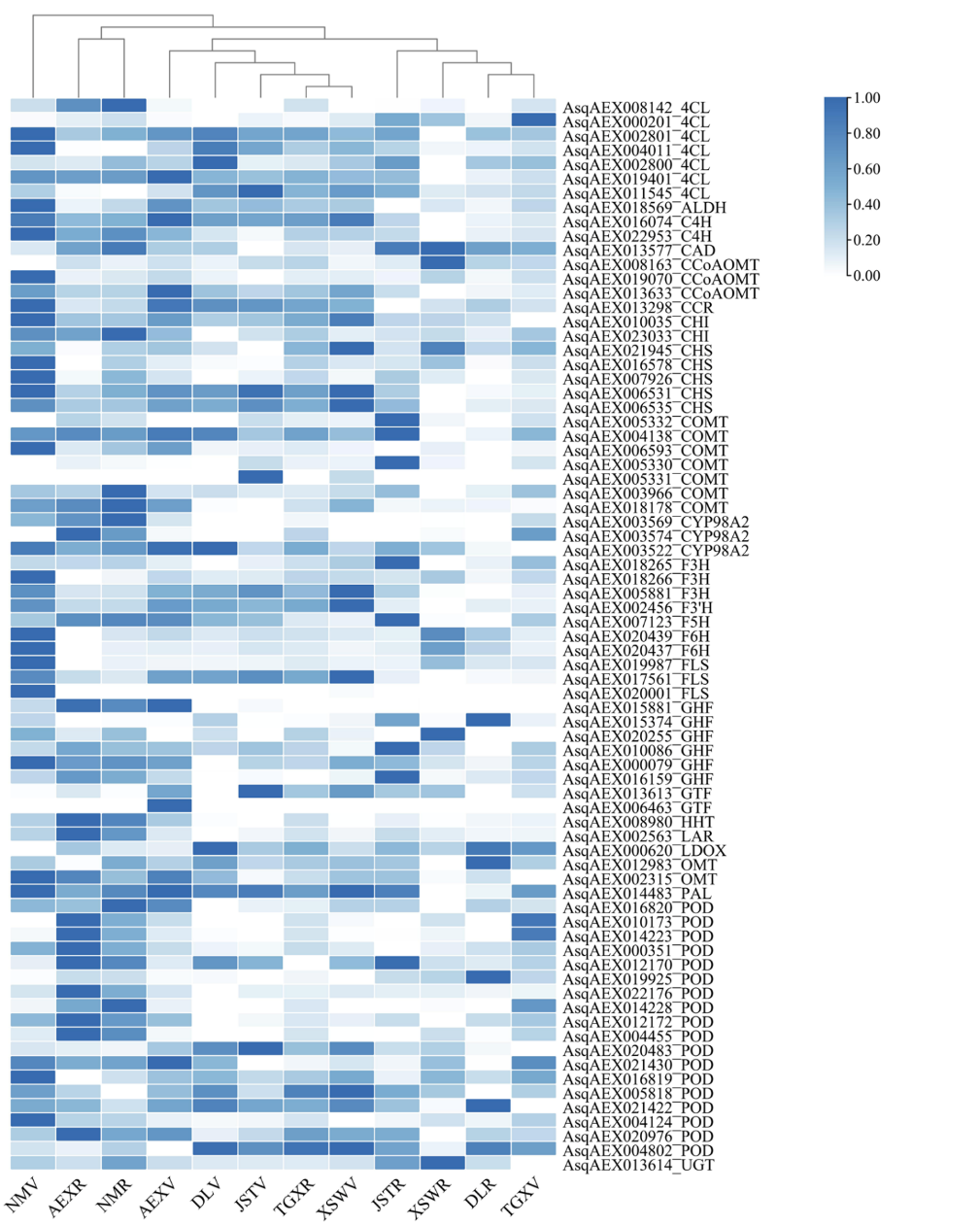
**

**Supplementary Figure 4.** Expression profile of flavonoids-related DEG. Heatmap was scaled by row and clustered by column. 4CL, 4-coumarate--CoA ligase; ALDH, Aldehyde dehydrogenase; C4H, Trans-cinnamate 4-monooxygenase; CAD, Cinnamyl alcohol dehydrogenase; CCoAOMT, Caffeoyl-CoA O-methyltransferase; CCR, Cinnamoyl-CoA reductase 1; CHI, Chalcone-flavanone isomerase; CHS, Chalcone synthase; COMT, Caffeic acid 3-O-methyltransferase; CYP98A2, cytochrome P450 98A2; F3H, Flavanone 3-dioxygenase; F3’H, Flavonoid 3'hydroxylase; F5H, cytochrome P450 84A1; F6H, Feruloyl-CoA 6-hydroxylase; FLS, Flavanol synthase; GHF, Glycosyl hydrolase family protein; GTF, Glycosyltransferase; HHT, Omega-hydroxypalmitate O-feruloyl transferase; LAR, Leucoanthocyanidin reductase; LDOX, Leucoanthocyanidin dioxygenase 2; OMT, O-methyltransferase; PAL, Phenylalanine ammonia-lyase; POD, Peroxidase; UGT, UDP-Glycosyltransferase superfamily protein.

| 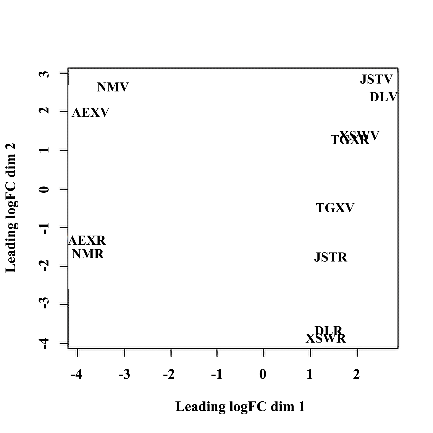 | 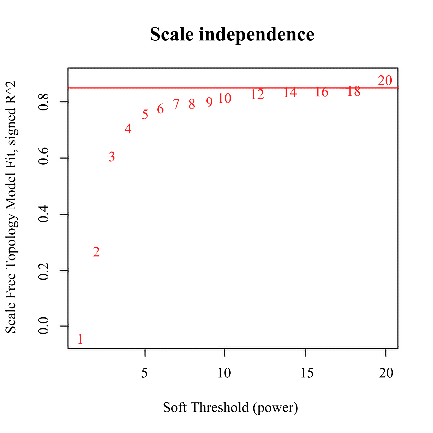 | 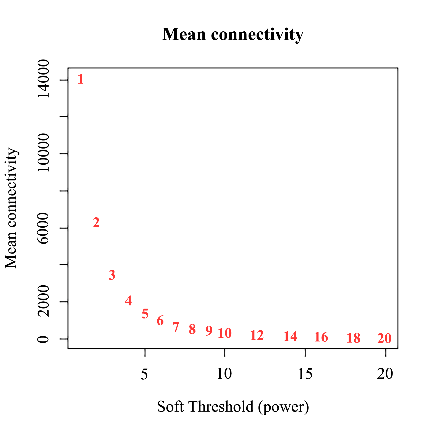 | 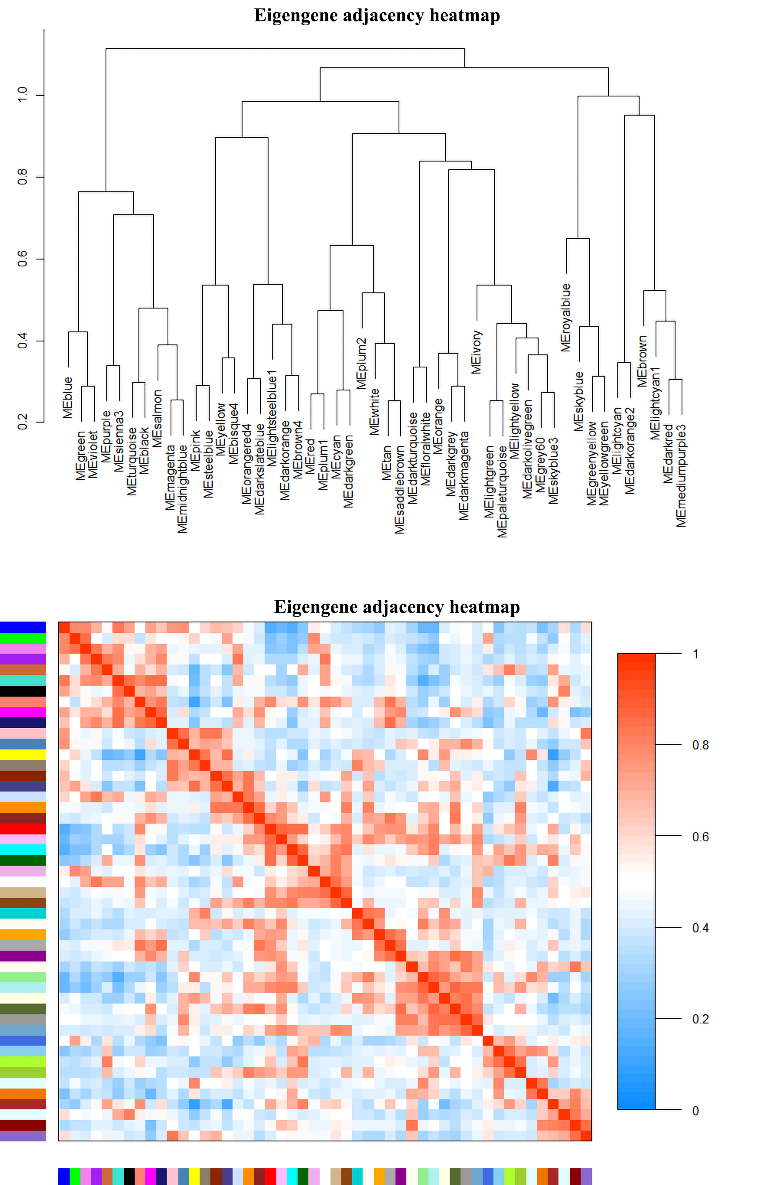 |
| --- | --- | --- | --- |
| (**A**) | (**B**) | (**C**) |  |
| 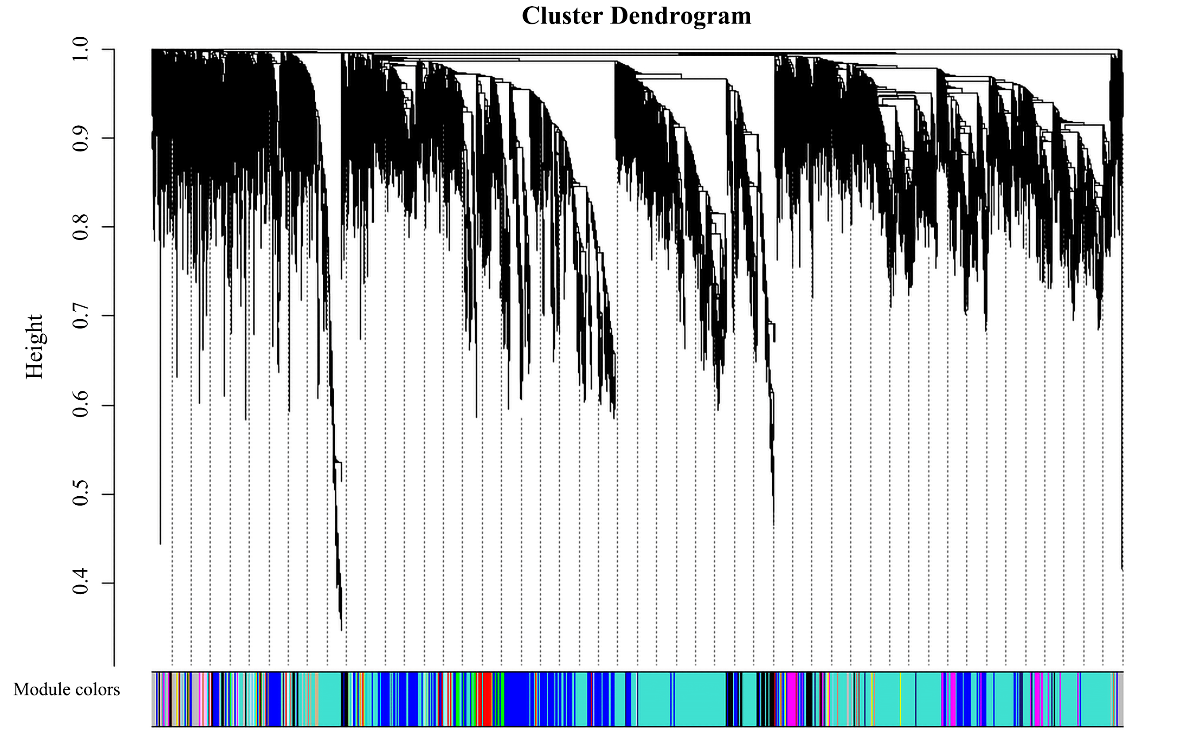 | | |  |
| (**D**) | | | (**E**) |

**Supplementary Figure 5.** Statistics of WGCNA. (**A**), PCA of the twelve tested samples; (**B**) soft-threshold selection; (**C**), mean connectivity; and (**D**), cluster dendrogram; (**E**), adjacency of eigengene modules.

**
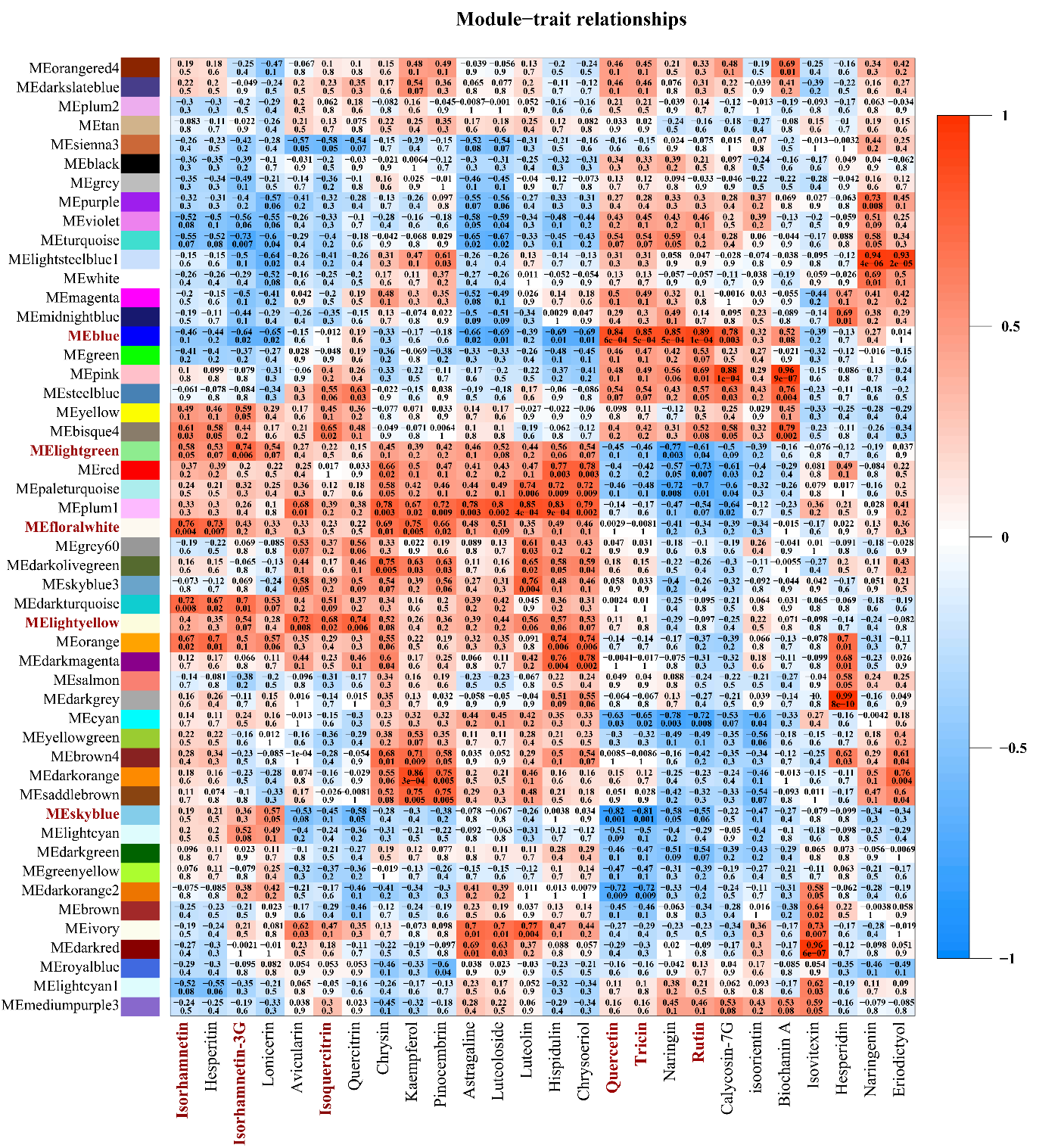
**

**Supplementary Figure 6.** Module-trait relationships between 50 gene modules and 26 flavonoids. Correlation and p-value of corresponding flavonoids and gene modules are shown as numbers up and down in each cell, respectively. The color scale on the right side represents module-trait correlation from −1 (blue) to 1 (red).

**
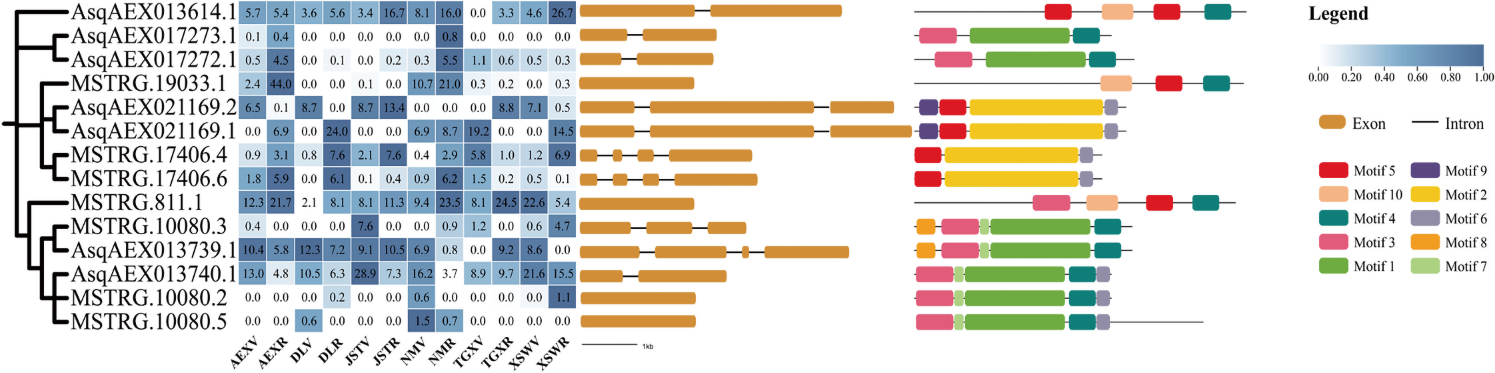
**

**Supplementary Figure 7.** *AsUGT78D2* gene family identification. From left to right, the four columns showed the phylogenetic tree, heatmap of expression profiles (TPM) in each of the twelve sample, gene structure, and conserved motifs visualization of the 14 candidates *AsUGT78D2*, respectively. Numbers in each cell of the expression profile heatmap were genes original TPM value. Introns were shown in equal length. Conserved motifs were shown as different colored bars, according to the actual length and position of peptides.

| **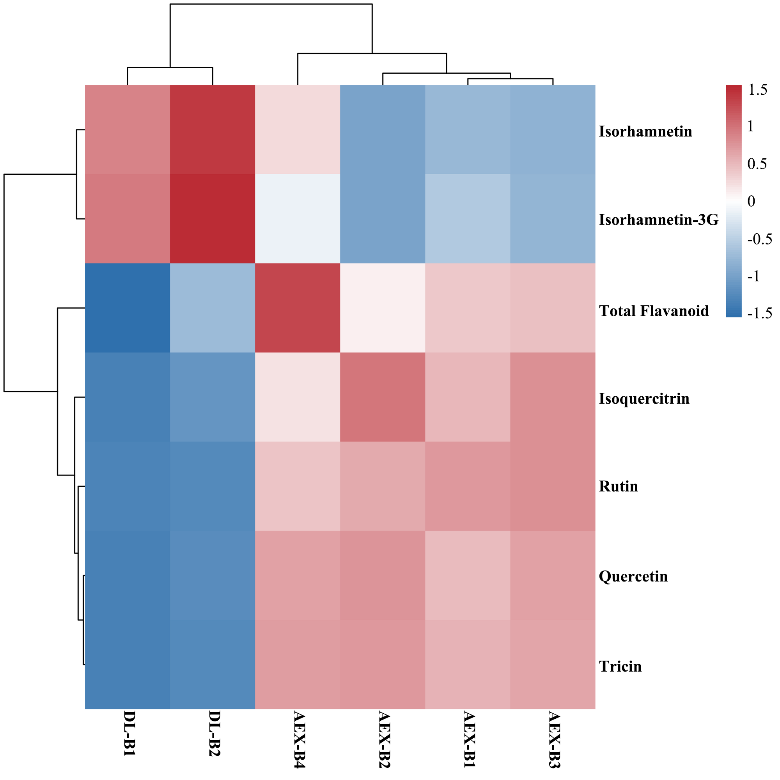**  (**A**) | **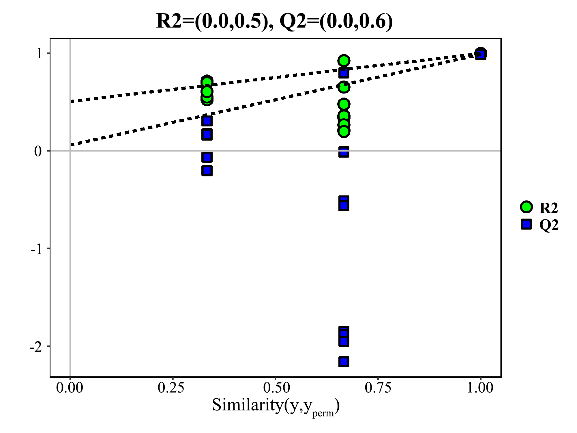** | (**B**) |
| --- | --- | --- |
|  | **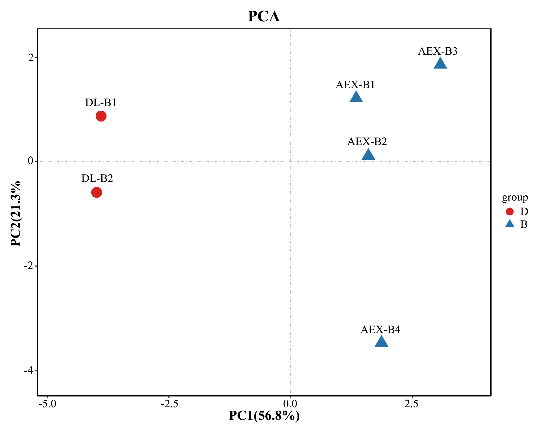** | (**C**) |

**Supplementary Figure 8.** Statistics of OPLS-DA between DLR and AEXR. (**A**), sample clustering heatmap, (**B**), permutation, and (**C**), PCA of samples in DLR and AEXR, respectively.

| **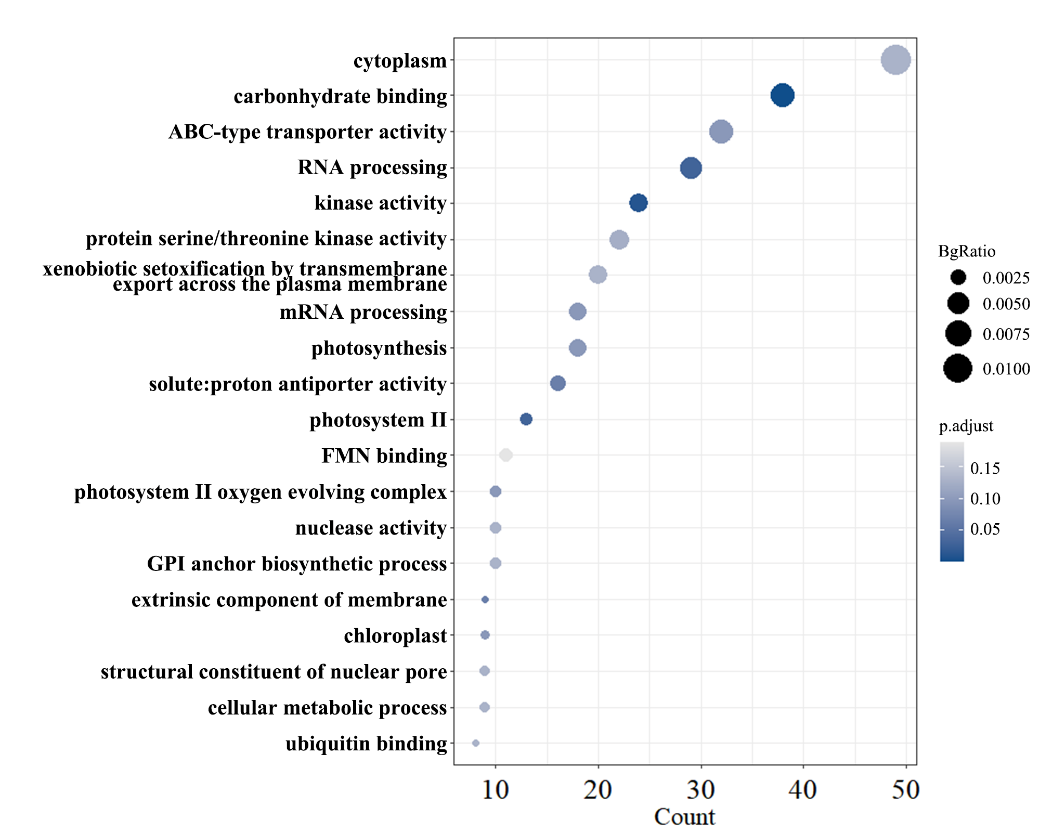** |
| --- |
| (**A**) |
| **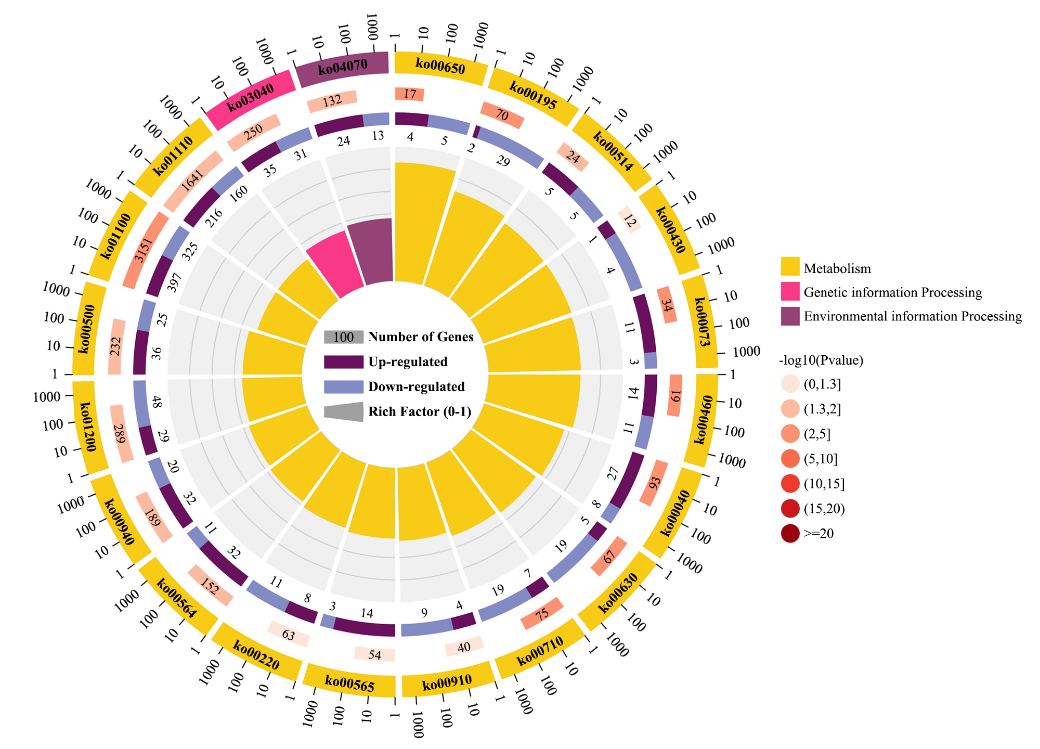** |
| (**B**) |

**Supplementary Figure 9.** GO (**A**) and KEGG (**B**) enrichment of DEG between DLR and AEXR.
